# *Puerariae lobatae* Radix ameliorates chronic kidney disease by reshaping gut microbiota and downregulating Wnt/β‑catenin signaling

**DOI:** 10.3892/mmr.2024.13241

**Published:** 2024-05-14

**Authors:** Peng Wu, Jingwen Xue, Zhangrui Zhu, Yao Yu, Qi Sun, Ming Xie, Benlin Wang, Pengcheng Huang, Zhengyuan Feng, Jie Zhao

**Affiliations:** 1 Department of Urology, Nanfang Hospital, Southern Medical University, Guangzhou, Guangdong 510515, P.R. China; 2 NMPA Key Laboratory for Research and Evaluation of Drug Metabolism, Guangdong Provincial Key Laboratory of New Drug Screening, School of Pharmaceutical Sciences, Southern Medical University, Guangzhou, Guangdong 510515, P.R. China

**Keywords:** *Puerariae lobatae* Radix, high salt diet, chronic kidney disease, Wnt/β-catenin pathway, gut microbiota, fecal microbiota transplantation

## Abstract

Gut microbiota dysfunction is a key factor affecting chronic kidney disease (CKD) susceptibility. *Puerariae lobatae* Radix (PLR), a traditional Chinese medicine and food homologous herb, is known to promote the gut microbiota homeostasis; however, its role in renoprotection remains unknown. The present study aimed to investigate the efficacy and potential mechanism of PLR to alleviate CKD. An 8-week 2% NaCl-feeding murine model was applied to induce CKD and evaluate the therapeutic effect of PLR supplementary. After gavage for 8 weeks, The medium and high doses of PLR significantly alleviated CKD-associated creatinine, urine protein increasement and nephritic histopathological injury. Moreover, PLR protected kidney from fibrosis by reducing inflammatory response and downregulating the canonical Wnt/β-catenin pathway. Furthermore, PLR rescued the gut microbiota dysbiosis and protected against high salt-induced gut barrier dysfunction. Enrichment of *Akkermansia* and *Bifidobacterium* was found after PLR intervention, the relative abundances of which were in positive correlation with normal maintenance of renal histology and function. Next, fecal microbiota transplantation experiment verified that the positive effect of PLR on CKD was, at least partially, exerted through gut microbiota reestablishment and downregulation of the Wnt/β-catenin pathway. The present study provided evidence for a new function of PLR on kidney protection and put forward a potential therapeutic strategy target for CKD.

## Introduction

Chronic kidney disease (CKD), a slowly progressive and irreversible kidney function failure, affects ~10% of the global population (1). Accumulating clinical evidence suggests that excessive sodium intake accelerates the progression of CKD and its related hypertension (2,3). Additionally, a high level of sodium has been proven to induce kidney cytokine expression as well as tissue regeneration and fibrosis by activating Wnt/β-catenin and TGF-β signaling (4–6). Consequently, excessive sodium intake is an important risk factor for CKD progression. Therefore, besides advocating salt reduction, it is critical to explore remedies to reconcile the adverse impact of excessive sodium consumption on CKD.

The intestine has a rich and structurally diverse microbiome that is engaged in multiple interactions affecting host health by influencing the intrinsic immunity and metabolism. Moreover, the composition and function of the gut microbiota is dynamic and easily affected by diet. There is an effect of dietary habits on host-microbe interactions, and nutrition can either maintain homeostasis or lead to disease susceptibility (7). Although salt is one of the most essential dietary components, excessive salt intake has been extensively studied and proven to cause a wide range of diseases through its effects on the gut microbiome (8). Previous studies have shown that high salt (HS) consumption results in alteration of the gut microbiota structure that may be pro-inflammatory, which exacerbates colitis and hypertension (9,10). In addition, HS-induced gut barrier disruption triggers renal function injury (11). These data indicated that excessive salt intake could cause susceptibility to a wide range of diseases through the gut microbiome. Moreover, depletion of the gut microbiome or administering probiotics can ease the progression of numerous diseases (11,12). Taken together, ideas were provided to reverse the progression of HS-related CKD through regulation of the gut microbiome.

A previous review advocated non-pharmacological strategies such as dietary and lifestyle modulation as well as kidney disease-specific pharmacological interventions to achieve kidney function preservation (13). *Puerariae lobatae* Radix (PLR) is rich in nutrients, including flavonoids, starch, saponins and amino acids, and has always been used as a medicine and food homologous herb to relieve gastrointestinal and cardiovascular diseases (14). Additionally, PLR is consistent with the principles of the CKD management guidelines that advocate dietary control (15). Multiple studies have indicated that PLR and its active ingredient might alleviate diabetes-related kidney damage and non-alcoholic fatty liver disease through modulating the gut microbiota (16,17). Previously, it was also confirmed that PLR promotes gut microbiota homeostasis, increases the relative abundance of beneficial bacteria, and protects the structural and functional integrity of the gut barrier, blood-brain barrier and placental barrier, thus affecting the symptoms of vascular anomaly-related diseases including ischemic stroke and pre-eclampsia (14,18). CKD is a disease closely related to host metabolic disorder and vascular dysfunction and bioflavonoids and other bioactive compounds have been verified to relieve CKD-associated biochemical abnormalities and kidney inflammation (19). However, few studies have focused on whether PLR exerts a protective effect on these conditions.

Because excessive sodium intake is a risk factor for the development of CKD, dietary preferences have great potential in influencing host health (11). In the present study, a HS-induced CKD murine model was constructed and the renoprotective effects of PLR were investigated.

## Materials and methods

### Source and preparation of PLR decoction

Medicinal slices form of PLR were purchased from Guangzhou Weida Company. Standard substance of puerarin (purity ≥99.0%), daidzin (purity ≥98.0%) and daidzein (purity ≥98.0%) were purchased from Chengdu Must Bio-Technology Co., Ltd. PLR decoction preparation was conducted as previously described (14,20,21). In brief, a certain quality of PLR was received and extracted in boiling distilled water at a ratio of 1:10 (w/v) for 1 h; the filtrate was collected and the residue was boiled with distilled water (1:6, w/v) for another 1 h. The aforementioned filtrates were concentrated and the content of puerarin, daidzin and daidzein was quantified by using ultra-performance liquid chromatography (specific method is listed in Table SI and Fig. S1). Briefly, the column was an Agilent ZORBAX SB-C18 (3.0×100 mm, 1.8-Micron). The mobile phase comprised methanol and water with a flow rate of 0.3 ml/min. Changes in the proportion of the mobile phase are shown in Table SI. Puerarin, daidzin and daidzein were detected at a UV wavelength of 250, 270 and 260 nm, respectively. Testosterone (240 nm) acted as the internal standard. The retention times of puerarin, daidzin and daidzein were 3.82, 5.19 and 9.55 min, respectively. Organic phase methanol was obtained from Tianjin Damao Chemical Reagent Factory. The respective concentrations of these three compounds were 10.9, 17.5 and 0.3 mg/ml.

### Animals and experimental design

All animal experimental procedures were conducted in strict accordance with the National Institute of Health guidelines and were approved (approval no. L-2019216) by the Institutional Animal Ethical Care Committee of Southern Medical University Experimental Animal Center (Guangzhou, China) (22). Specific pathogen-free male C57BL/6 mice (6–8 week, weighing 20–26 g) were purchased from SPF Biotechnology Co., Ltd. The total number purchased was 55. All mice were housed under standard conditions of temperature (22±1°C) and humidity (50±5%) with a 12/12-h light-dark cycle and free access to food and water. After 1 week of acclimatization, HS-feeding mice were administered drinking water containing 2% (w/v) NaCl for 8 weeks as previously described (11) (HS, n=6), The amount of drinking water provided minus the amount of water remaining indicated the amount of water consumed during the day. while PLR-intervention mice received *Pueraria* decoction (containing 18, 36 and 72 mg/kg Puerarin, respectively) by oral gavage once a day along with 8 weeks HS feeding (PLR-L, PLR-M and PLR-H, n=6 per group). Control mice received normal drinking water during the experiment (CON, n=10). There were no differences in baseline covariates among groups. The feces and urine were collected every week for further examination. After an 8-week period of modeling, cervical dislocation was performed under complete anesthesia with an intraperitoneal injection of 1.5% sodium pentobarbital at a concentration of 40 mg/kg. Death of mice was verified by cardiac arrest, a reduction in body temperature, and a lack of response to strong stimuli. Sampling operation time was ~10–15 min per mouse. Serum, kidney, liver, spleen, colon and cecal contents samples were harvested in a sterile manner for further analysis. Calculation of organ index was performed using organ weight/body weight ratio. After separation of the colon, the length was measured from the junction of the cecum and colon to the anal canal, which was recorded as the length of the colon. Blood collection (0.5–0.8 ml) was performed by open laparotomy through the abdominal aorta with a fine needle after euthanasia under complete anesthesia.

For blood pressure measurement, systolic blood pressure (SBP) and mean blood pressure (MBP) were measured every week via non-invasive tail cuff method, using a BP-2010A instrument (Beijing Softron Biotechnology Co., Ltd.). All measurements were operated between 8 to 12 am. At least six continuous stable results of each mouse were obtained and the average value was calculated as the final result.

The mice were weighed at regular times each day, their bedding was changed and their health was assessed. Humane endpoints were as follows: The body weight of mice being 20% lower than before the experiment, mice being unable to feed or drink, or not responding to gentle stimulation. In the aforementioned circumstances, mice would be considered unsuitable for further experimentation and would be euthanized by neck dissection after complete anesthesia using the anesthesia method and dosage as aforementioned. In the present study, none of the mice reached these humane endpoints.

### Fecal microbiota transplantation (FMT) experiment

FMT experiment was performed based on the modified method previously described (11,23). In brief, feces from the donor mice (HS and PLR-H group mice) were collected in aseptic centrifugal tubes respectively and resuspended in PBS at 125 mg/ml, the mixtures were centrifuged at 1,000 × g for 1 min at 37°C and the supernatants were collected and saved in separate 1.5 ml tubes for subsequently microbiota transplantation. Before FMT experiment, the acceptor male C57BL/6 mice (6–8 weeks) were given antibiotics (vancomycin, 100 mg/kg; neomycin sulfate 200 mg/kg; metronidazole 200 mg/kg; and ampicillin 200 mg/kg) intragastrically once a day for 1 w to deplete the gut microbiota. All the mice then received drinking water containing 2% (w/v) NaCl for 8 weeks as aforementioned. A volume of 150 µl of the fecal microbiota solution was simultaneously orally gavaged to mice once a day in the first 2 weeks, every other day in the following 2 weeks and twice each week in the last 4 weeks. The mice that received fecal microbiota solution from HS mice or PLR-H mice were referred to as HS-FMT group or PLR-FMT group (n=8 per group). Control mice received normal drinking water during the experiment (CON, n=5). The total number of mice purchased in this part of the experiment was 21. Blood pressures were measured as aforementioned every week. All the mice had free access to food and water, and the feces and urine were collected before sacrifice. Mice were sacrificed under anesthesia and then blood, kidney, liver, spleen, colon and cecal contents samples were harvested in a sterile manner for further analysis.

### 16S rDNA gene sequencing and microbe analysis

Feces were collected in sterilized 1.5 ml tubes and frozen at −80°C before DNA extraction. Microbial DNA from fecal samples were extracted as previously described (24,25). V3-V4 region of 16S rDNA was amplified by PCR using the following primers: 341F, 5′-CCTACGGGNGGCWGCAG-3′ and 806R, 5′-GGACTACHVGGGTATCTAAT-3′. Amplicons were purified using the AxyPrep DNA Gel Extraction Kit (cat. no. AP-GX-250; Axygen; Corning, Inc.). Equimolar pooling of purified amplicons was performed and paired end sequenced (PE250) was conducted on an Illumina platform according to the standard protocols. Alpha diversity were calculated in QIIME (26) (version 1.9.1). The abundance statistics of each taxonomy was visualized using Krona (27) (version 2.6). Principal coordinate analysis (PCoA) and non-metric multidimensional scaling (NMDS) of weighted unifrac distances were generated in R project Vegan package (version 2.5.3). Biomarker features in each group were screened by LEfSe software (28) (version 1.0). Microbial dysbiosis index (MDI) was calculated as follows: MDI=log_10_[(total abundance in genera increased in disease group)/(total abundance in genera decreased in disease group)]. The Kyoto Encylopedia of Genes and Genomes (KEGG) pathway analysis of the operational taxonomic units was inferred using Tax4Fun (29) (version 1.0) and was generated using Omicsmart, a dynamic real-time interactive online platform for data analysis (http://www.omicsmart.com). Spearman correlation coefficient between environmental factors and species was calculated in R project psych package (version 1.8.4) then generated using the Wekemo Bioincloud (https://www.bioincloud.tech). The calculated P-value was conducted through false discovery rate (FDR) correction, taking FDR ≤0.05 as a threshold.

### RNA-seq analysis

Kidney samples were collected in sterilized 1.5-ml tubes and frozen at −80°C before DNA extraction. Total RNA was extracted using the Eastep™ Super Total RNA Extraction Kit (cat. no. LS1040; Promega Corporation) according to the manufacturer's instructions. After total RNA was extracted, eukaryotic mRNA was enriched by Oligo(dT) beads. Then fragments were transcribed into cDNA by using NEBNext Ultra RNA Library Prep Kit for Illumina (cat. no. 7530; New England Biolabs, Inc.). The ligation reaction was purified, and PCR amplified. The resulting cDNA library was sequenced using Illumina Novaseq6000 by Gene Denovo Biotechnology Co. Differential expression analysis of RNAs was performed by DESeq2 (30) software between two different groups. The transcripts with the parameter of FDR below 0.05 and absolute fold change ≥2 were considered differentially expressed transcripts. Gene set enrichment analysis (GSEA) was performed using software GSEA (31) and MSigDB (31) to identify whether a set of genes in specific KEGG pathway shows significant differences in two groups.

### Gene expression analysis

Total RNA was extracted from kidney and colon tissue using the Animal Total RNA Isolation Kit (cat. no. RE-03011/03014; Foregene Co., Ltd,) according to the manufacturer's instructions. A reverse transcription enzyme, HiScript III RT SuperMix for qPCR (+gDNA wiper) (Vazyme Biotech Co., Ltd.), was applied to obtain cDNA (temperature and duration: 50°C for 15 min; 85°C for 5 sec). The quantitative PCR reactions (initial denaturation: 95°C for 30 sec; 40 cycles of amplification at 95°C for 10 sec and 60°C for 30 sec; followed by melting curve analysis at 95°C for 15 sec, 60°C for 1 min and 95°C for 15 sec) were performed on the LightCycler480 (Roche Diagnostics) using a ChamQ SYBR qPCR Master Mix (Vazyme Biotech Co., Ltd.). Relative quantification of target genes were calculated using the 2^−ΔΔCq^ method (32). GAPDH was used as a control gene. All the target gene primer sequences are listed in Table SII.

### Protein expression and biochemical analysis

The kidney and colon tissue protein were extracted with a commercial RIPA lysis buffer (cat. no. PC101; Epizyme Biomedical Technology Co., Ltd.) supplemented with 1% cocktail protease inhibitor (cat. no. MB2678; Dalian Meilun Biology Technology Co., Ltd.). Protein samples were quantified using a BCA Protein Assay Kit (cat. no. ZJ102; Epizyme Biomedical Technology Co., Ltd.) and 40 µg protein was loaded per lane. Samples were separated by SDS-PAGE on 10% gels and transferred to a PVDF membrane. After blocking with 5% non-fat milk for 1 h at 37°C, samples were incubated overnight at 4°C with the following primary antibodies: Rabbit anti-Occludin (1:2,000; cat. no. ab216327; Abcam), rabbit anti-ZO-1 (1:2,000; cat. no. ab216880; Abcam), rabbit anti-Claudin-1 (1:2,000; cat. no. YT0942; ImmunoWay Biotechnology Company), mouse anti-TNF-α (1:1,000; cat. no. YM3477; ImmunoWay Biotechnology Company), rabbit anti-IL-1β (1:1,000; cat. no. YT5201; ImmunoWay Biotechnology Company), rabbit anti-β-catenin (1:5,000; cat. no. 51067-2-AP; Proteintech Group, Inc.) and mouse anti-β-actin (1:5,000; cat. no. RM2001; RayBiotech, Inc.). Subsequently, they were incubated with horseradish peroxidase-conjugated goat anti-mouse/rabbit secondary antibody at 37°C for 1 h (1:5,000; cat. no. LF101/LF102; Epizyme Biomedical Technology Co., Ltd.). Membranes were visualized with a Femto Light Chemiluminescence Kit (cat. no. SQ201; Epizyme Biomedical Technology Co., Ltd.) and imaged with a gel image processing system (cat. no. 92-15313-00; FluorChem R System; Bio-Techne). The band intensity was assessed using ImageJ software (version 1.53K; National Institutes of Health).

Serum creatinine was determined by using a Creatinine Assay kit (sarcosine oxidase) (cat. no. C011-2-1; Nanjing Jiancheng Bioengineering Institute). The urinary protein, serum lipopolysaccharide (LPS) and serum TNF-α level were detected manually with commercial ELISA Kits (cat. nos. MM-44286M2, MM-0634M2 and MM-0132M2; Shanghai Meimian Biotechnology, Co., Ltd.) according to the manufacturer's protocols. Serum and urine K^+^, Ca^2+^ and Na^+^ concentrations were determined on an automatic biomedical analyzer (Roche Diagnostics).

### FD-4 permeability experiment

FITC Dextran 4-KD (FD-4; Sigma-Adrich; Merck KGaA) was used to detect intestinal permeability *in vivo*. FD-4 was orally administered to mice at a dose of 0.6 mg/kg 4 h before serum was harvested. The FD-4 level in serum was detected in the dark, based on a standard curve and spectro-fluorometrically with an excitation wavelength of 485 nm and an emission wavelength of 530 nm in a microplate fluorescence reader (Infinite^®^ M1000 PRO; Tecan Group, Ltd.).

### Imaging of intestinal inflammation in vivo

L-012 (FUJIFILM Wako Pure Chemical Corporation) was used to confirm intestinal inflammation *in vivo* (33). Mice were anesthetized with 3–4% isoflurane induction by inhalation and maintained with 1–1.5% isoflurane and injected with 100 µl of a 20-mmol L-012 solution. After 1 min, IVIS Spectrum CT system was used to acquire bioluminescent images. For post-acquisition analysis, the Living Image software was used to quantify the bioluminescent signals at standardized regions of interest (ROIs) defined on the abdomen.

### Histological staining and analysis

The left kidney and colon tissue of mice were collected and fixed in 4% paraformaldehyde for 3 days at room temperature. The samples were then embedded in paraffin, sectioned at 4-µm thickness and stained with hematoxylin and eosin (H&E) (hematoxylin for 4 min and eosin for 20 sec) or Masson's trichrome (MASSON) (Weigert's iron hematoxylin stain for 8 min, ponceau S for 5 min and aniline blue for 2 min) at room temperature. The kidney tissue histological damage was assessed according to a previously described scoring method. Briefly, the extent of damage was assessed based on interstitial inflammatory cell infiltration, tubular pattern of renal tubules and loss of brush border. The lesion score was quantified as follows: score 0=0%; score 1=1–10%; score 2=11–25%; score 3=26–75%; score 4=76–100% (34). The kidney fibrosis area was selected randomly from at least 5 points of cortical fields and quantified using ImageJ software (version 1.53K; National Institutes of Health). Immunohistochemistry was performed using the primary antibodies for Occludin, ZO-1 and Claudin-1. Quantification of the average optical density was performed by automated image analysis in five randomly chosen fields of each sample (magnification, ×200). All images were scanned by a NanoZoomer Digital slide scanner and captured at ×200 or ×400 magnification with an NDP. View2 Plus Image viewing software (Hamamatsu Photonics K.K.) was used.

### Immunofluorescence

Colon paraffin section samples were generated as aforementioned. TBS solution containing 0.3% Triton X-100 (cat. no. 9002-93-1; Beijing Solarbio Science & Technology Co., Ltd.), 0.25% protein free rapid blocking buffer (cat. no. PS108P; Epizyme Biomedical Technology Co., Ltd.) and 5% goat serum (cat. no. BL210A; Biosharp Life Sciences) was used to block samples for 1 h at room temperature. The samples were then incubated at 4°C with rabbit anti-β-catenin antibody (1:50; cat. no. 51067-2-AP; Proteintech Group, Inc.) overnight. Subsequently, they were incubated with Alexa Fluor 488-labeled goat anti-rabbit secondary antibody (1:500; cat. no. A0423; Beyotime Institute of Biotechnology) at 37°C for 1 h. Then the slices were washed and stained with DAPI (0.5 µg/ml) for 10 min. Images were captured with fluorescent microscopy (Olympus Corporation). Quantification of the fluorescence intensity was calculated by automated image analysis in five randomly chosen fields of each sample (magnification, ×200).

### Statistical analysis

Results were represented as the mean ± standard error of the mean (SEM) and performed using GraphPad Prism software (version 8.0) (GraphPad Software; Dotmatics). When comparing the difference in means between two samples, the Mann-Whitney test was used for non-normal distributions; when comparing the difference in means between multiple samples, the one-way ANOVA was used if the data were normally distributed and the variance was homogeneous, and the Kruskal-Wallis test was used for non-normal distributions. Comparisons between groups involving the same time point and between groups at different time points within the same group were made using two-way repeated-measures ANOVA. Dunn's post hoc test was used after Kruskal-Wallis test, and Bonferroni's post hoc test or Tukey's HSD post hoc test was used after ANOVA. Adonis analysis and anosim analyses were used for PCoA. P<0.05 was considered to indicate a statistically significant difference.

## Results

### PLR relieves elevated SBP and slow weight gain induced by an HS diet

**Figure 1.**
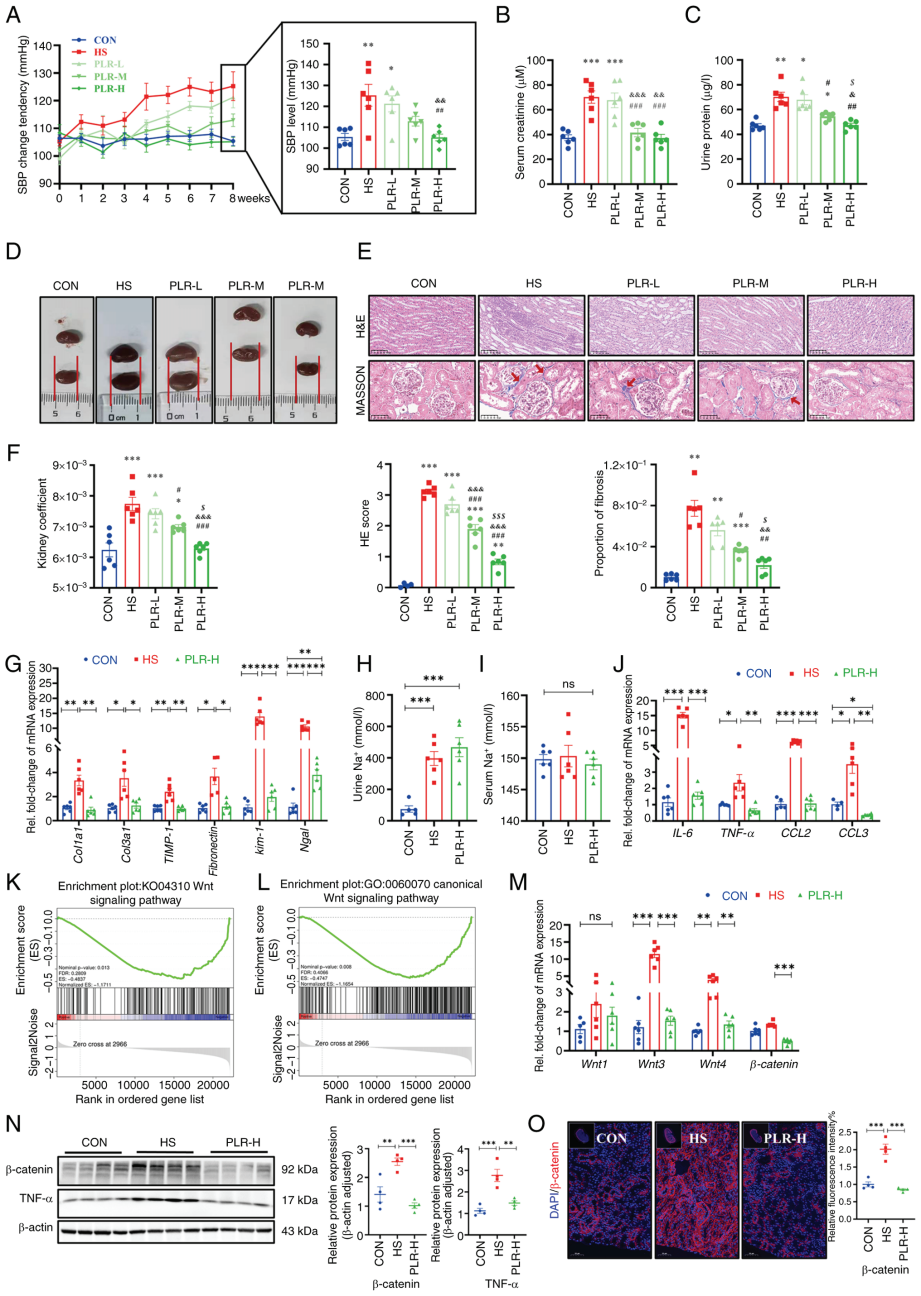
PLR alleviates kidney injuries induced by HS diet and downregulates the Wnt/β-catenin pathway in kidney of HS mice. (A) SBP change of groups were evaluated non-invasively in the course of experiment (n=6). (B) Serum creatinine after 8 weeks (n=6). (C) Urine protein after 8 weeks (n=6). (D) Representative gross anatomy images of the kidney (n=6). (E) H&E and MASSON staining of the kidney and the pathology scores and histological analysis. Fibrosis in the glomeruli and renal interstitium is indicated with red arrows. H&E staining scale bar, 100 µm; MASSON staining scale bar, 50 µm (n=6). (F) Kidney coefficient (n=6). (G) RT-qPCR analysis of kidney injuries-related genes (n=6). (H) Urine Na^+^ concentration (n=6). (I) Serum Na^+^ concentration (n=6). (J) RT-qPCR analysis of inflammatory factors-related genes in kidney (n=6). (K) Gene-set enrichment analyses based on Kyoto Encyclopedia of Genes and Genomes database performed on the RNA sequencing data (n=3). (L) Gene-set enrichment analyses based on Gene Ontology database performed on the RNA sequencing data (n=3). (M) RT-qPCR analysis of Wnt1, Wnt3, Wnt4 and β-catenin genes in kidney (n=5-6). (N) β-catenin and TNF-α protein levels in the kidney (n=4). (O) Representative *in situ* detection of β-catenin in renal cortex was measured by immunofluorescent staining. Kidney tissue sections were stained with DAPI (blue) and probed with β-catenin (red). Scale bar, 50 µm (n=4). Results are expressed as the mean ± SEM. *P<0.05, **P<0.01 and ***P<0.001 vs. the CON group; ^#^P<0.05, ^##^P<0.01 and ^###^P<0.001 vs. the HS group; ^&^P<0.05, ^&&^P<0.01 and ^&&&^P<0.001 vs. the PLR-L group; ^$^P<0.05, and ^$$$^P<0.001 vs. the PLR-M group in A-E. ***P<0.001, **P<0.01 and *P<0.05 were determined by one-way ANOVA with Bonferroni's post hoc test in G-J and M-O. PLR, *Puerariae lobatae* Radix; HS, high salt; SBP, systolic blood pressure; H&E, hematoxylin-eosin; RT-qPCR, reverse transcription-quantitative PCR; ns, not significant.

There were no differences in the baseline weight and blood pressure among the groups (Fig. S2A-C); however, after consuming the same amount of sodium, there was a significant difference in SBP among the groups (Fig. 1A). Compared with the CON group, the SBP of the HS group showed significant elevation from the 5th week. By contrast, the SBP of the PLR-H group maintained no difference from the CON group during the whole experiment. SBP in the PLR-L and PLR-M groups were lower than the HS group; however, this reduction was not significant at the end of the experiment (Fig. 1A). Additively, changes in MBP were not significantly different among the groups (Fig. S2D). These results suggested that high-dose PLR supplementation could effectively reduce SBP in CKD. Besides, the weight change was also recorded; HS group gained weight more slowly and exhibited significant different in weight compared with those in the CON group from the 4th week onwards. PLR intervention could maintain a normal rate of weight gain to some extent (Fig. S2E).

### PLR alleviates CKD and renal fibrosis induced by an HS diet

In the 8th week, serum creatinine, urine protein and kidney histologic stains were used to assess the extent of kidney damage as well as the efficiency of PLR on kidney protection. Compared with the CON group, the HS group revealed significantly aggravated serum creatinine and urine protein levels (Fig. 1B and C); however, these biochemical parameters were significantly improved in the PLR-M and PLR-H groups. Moreover, the macroscopic images of the kidneys of the PLR-M and PLR-H groups exhibited markedly less hyperemia and swelling compared with those of the HS and PLR-L groups (Fig. 1D). Consistent with this appearance, the kidney coefficient and the histopathological injury observed by H&E staining and the proportion of fibrosis observed by MASSON staining were significantly lower in the PLR-M and PLR-H groups than those in the HS group (Fig. 1E and F). Such histological coefficient differences were not indicated in the liver and spleen among the groups (Fig. S2F and G), however. Notably, middle-and high-dose PLR supplementation significantly alleviated CKD. PLR-H more efficiently reduced the degree of fibrosis and renal H&E scores compared with PLR-M. Taken together, the high PLR dose was considered as the most effective for CKD protection and was evaluated in the following experiment.

Compared with the CON group, the HS mice demonstrated significantly upregulated mRNA levels of *Col1a1, Col3a1, TIMP-1* and *Fibronectin* (Fig. 1G), which indicated fibrosis in the kidneys of the HS group. High-dose PLR supplementation significantly downregulated these fibrosis-related gene expression levels. In addition, the mRNA levels of *Kim-1* and *Ngal*, which indicated renal tubular injury, were significantly higher in the HS group than in the PLR-H group. Considering that the amount of salt intake could influence kidney injury, the total water intake was compared and it was found that the HS and PLR (all doses) groups consumed similar amounts of salt in the whole experiment (Fig. S2H). Additionally, both HS intervention and administation of high-dose PLR did not alter serum sodium loading, but they did increase urinary sodium excretion, as measured by urine and serum Na^+^ (Fig. 1H and I). Serum concentrations of the other major ions also had no differences among the groups (Fig. S2I and J).

### PLR mitigates the inflammatory response and downregulates the Wnt/β-catenin pathway in the kidney

To investigate the mechanism by which high-dose PLR exerted its renoprotective effects, RNA-seq was performed to assess the gene expression profile of the kidneys in the HS group vs. the PLR-H group. Compared with the HS group, administration of high-dose PLR altered the expression of 532 genes in the kidneys, with 53 transcripts being significantly upregulated and 479 being downregulated (Fig. S2K). GSEA based on the Gene Ontology (GO) and KEGG databases were performed on the RNA-seq data and revealed that a high PLR dose substantially decreased the expression of genes related to the canonical Wnt signaling pathway (Fig. 1K and L). Furthermore, it was found that HS intake induced a significant increase in the mRNA levels of IL-6, TNF-α, CCL2 and CCL3 in the kidney (Fig. 1J), and this effect was reversed in the PLR-H group. To further verify whether the protective effect of high-dose PLR is related to the Wnt/β-catenin pathway, the mRNA levels of molecules in this pathway were next analyzed. As presented in Fig. 1M, HS intake significantly increased the Wnt3 and Wnt4 mRNA levels. Furthermore, the protein level of β-catenin was measured (Fig. 1N and O), the expression level of which was significantly increased in the HS mice compared with the CON group, demonstrating that HS intake could activate the canonical Wnt signaling pathway. While high-dose PLR administration could significantly downregulate the mRNA and protein level of β-catenin, it also downregulated the mRNA levels of Wnt3 and Wnt4. In addition, TNF-α, which was able to induce β-catenin activation, was also found to have an elevated protein level in the kidney tissue in the HS group and was reduced by high-dose PLR administration. Overall, it was found that high-dose PLR could exert nephroprotective effects by reducing the activation of the Wnt/β-catenin pathway.

### PLR reduces intestinal inflammation and protects against intestinal barrier damage

**Figure 2.**
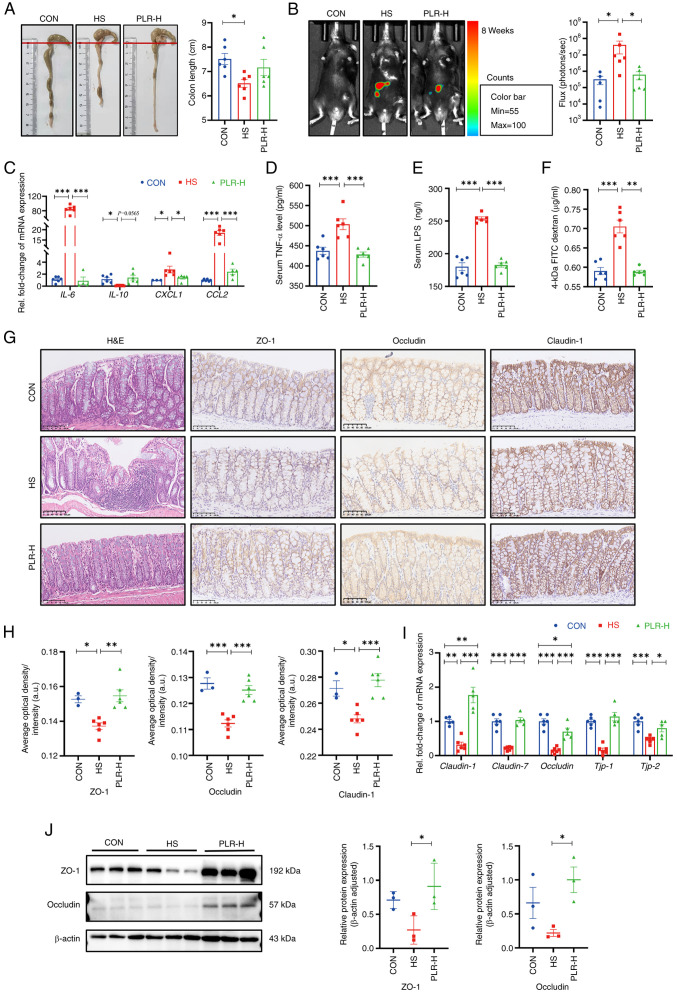
PLR reduces intestinal inflammation and protects against intestinal barrier damage in HS mice. (A) Representative gross anatomy images of the colon and the colon length measurement (n=6). (B) Representative L-012 fluorescent staining and animal fluorescence imaging (n=5-6). (C) RT-qPCR analysis of inflammatory factors' genes in colon (n=6). (D) Relative serum TNF-α levels (n=4-6). (E) Relative serum LPS levels (n=6). (F) FITC-dextran 4-KD level in the plasma (n=6). (G) H&E staining and ZO-1, Occludin and Claudin-1 immunohistochemical staining in colon tissues. Scale bar, 100 µm (n=3-6). (H) Quantification of immunohistochemistry (n=3-6). (I) RT-qPCR analysis of tight junction proteins genes in colon (n=5-6). (J) ZO-1 and Occludin protein levels in the colon (n=3). Results are expressed as the mean ± SEM. ***P<0.001, **P<0.01 and *P<0.05 were determined by one-way ANOVA with Bonferroni's post hoc test. PLR, *Puerariae lobatae* Radix; HS, high salt; RT-qPCR, reverse transcription-quantitative PCR; LPS, lipopolysaccharide; H&E, hematoxylin-eosin; ZO-1, zonula occludens 1.

Maternal intestinal damage, including inflammation and concomitant increased gut permeability, are involved in numerous extraintestinal diseases including CKD (35). Hence, the intestinal alterations were examined, as shown in Fig. 2A. Compared with the HS group, colon shortening was relieved to certain extent in the PLR-H group, although no statistical difference was observed. To further assess the inflammation level, pro-inflammatory cytokines in the colonic tissues were measured (Fig. 2C). The mRNA levels of IL-6, CXCL1 and CCL2 in the HS group were significantly higher, whereas the mRNA level of inflammatory protective factor IL-10 was reduced compared with those in the CON group. However, high-dose PLR supplementation significantly inhibited the expression of pro-inflammatory factors. In Fig. 2B, an L-012 chemiluminescent probe was used to assess intestinal reactive oxygen species (ROS) *in vivo* (33). The images indicated that chronic HS intervention markedly elevated the ROS concentration in the colon of mice, with a significantly increased total flux in photons, and this elevation was significantly dampened by high-dose PLR administration.

To further demonstrate the role of PLR administration in intestinal protection, intestinal tight junction markers in the colon were measured. Compared with the CON group, the HS group revealed significantly downregulated mRNA levels of tight junction proteins (Fig. 2I); whereas the serum TNF-α level, LPS level and FD-4 permeability were elevated (Fig. 2D-F). Moreover, the HS mice presented significantly deteriorative histopathological injury in the colon as observed by H&E staining, as well as decreased mRNA and protein levels of ZO-1, Occludin and Claudin-1 observed by immunohistochemical staining and western blotting (Fig. 2G-J). However, all the aforementioned damages induced by HS were largely attenuated by high-dose PLR administration. Collectively, the aforementioned results demonstrated that therapeutic high-dose PLR administration alleviated intestinal inflammation and reinforced the gut barrier.

### PLR reverses gut microbial dysbiosis and increases the relative abundance of beneficial bacteria

**Figure 3.**
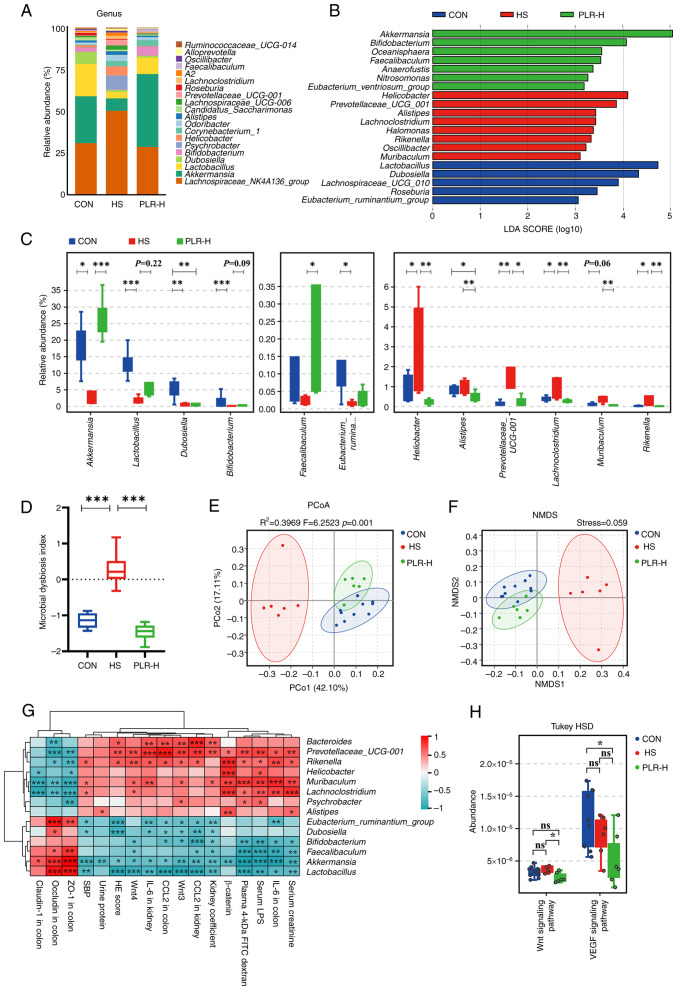
PLR reverses intestinal microbial dysbiosis in HS mice and remodels gut microbiota by increasing the relative abundance of probiotics. (A) Relative bacterial abundance at the genus level in the feces of mice. (B) Histogram of the LDA score showing the biomarker at the genus level of each group. (C) Relative abundance of indicator species at the genus level showing the enriched bacteria in the gut microbiome among groups. (D) Microbial Dysbiosis index of each group. (E) PCoA based on the weighted UniFrac analysis of operational taxonomic units. (F) NMDS based on the weighted UniFrac analysis of operational taxonomic units. (G) Correlation heatmap of major indicator species and biomarkers based on LDA score and major injury indicators, scale shows correlation coefficient. (H) Kyoto Encyclopedia of Genes and Genomes pathway analysis of function distribution and difference analysis based on Tax4Fun prediction results. Results are expressed as the mean ± SEM (n=6–10 for each group). ***P<0.001, **P<0.01 and *P<0.05 were determined by one-way ANOVA with Bonferroni's post hoc test or Kruskal-Wallis test with Dunn's post hoc test in C and D, adonis analysis and anosim analysis in E, Spearman analysis in G and ANOVA test with Tukey's HSD test in H. PLR, *Puerariae lobatae* Radix; HS, high salt; LDA, linear discriminant analysis; PCoA, principal coordinates analysis; NMDS, non-metric multidimensional scaling; ns, not significant.

A pathophysiological colon change is always associated with enteric dysbiosis (36,37). The impact of high-dose PLR on the gut microbiota composition and equilibrium was further examined via 16S rDNA sequencing. The relative abundance of Firmicutes as well as the Firmicutes/Bacteroidetes ratio in the cecal content of HS mice were lower than those in the cecal content of CON mice, whereas the *Bacteroidetes* level was significantly higher in the HS group (Fig. S3A). High-dose PLR intervention reversed the dominant position of Bacteroidetes and upregulated the Firmicutes/Bacteroidetes ratio. In addition, the bacterial composition in the cecal content samples from the three groups in terms of bacterial phyla and genera were significantly different (Figs. 3A and S3B). At the phylum level, Verrucomicrobia was the predominant phylum in the PLR-H group compared with that of the HS group. At the genus level, the fecal microbiota was dominated by *Lachnospiraceae*_NK4A136_group, *Akkermansia* and *Lactobacillus*. Besides, different abundances of fecal bacterial taxa in the three groups were identified by LEfSe analysis (Fig. 3B), which indicated that seven bacterial genera including short-chain fatty acid (SCFA)-producing bacteria, such as *Akkermansia* and *Bifidobacterium*, were enriched by PLR-H, while the other eight taxa, including *Rikenella, Prevotellaceae*_UCG-001 and *Lachnoclostridium* were enriched in the HS group (LDA score >3). Moreover, indicator species analysis showed a statistically significant higher relative abundance of *Faecalibaculum* and *Akkermansia* at the genus level in the PLR-H group than in the HS group (Fig. 3C). It was also found that Lactobacillus and Bifidobacterium showed an increasing trend after high-dose PLR administration compared with the HS group. Additionally, the MDI revealed an increased value in HS mice (Fig. 3D), which was reversed by high-dose PLR intervention, indicating a decrease in the level of intestinal flora disruption.

In the present study, the alpha diversity was impacted by neither HS nor PLR-H administration (Fig. S3C). However, NMDS and PCoA demonstrated that the overall structure of the gut microbiota was significantly different among the three groups (Fig. 3E and F), indicating that the gut microbiota structure in HS mice was markedly influenced and shifted closer towards that of the CON group by PLR intervention.

To further the present investigation, Spearman correlation analysis was performed (Figs. 3G and S3D). The relative abundances of *Akkermansia, Lactobacillus* and *Bifidobacterium* were negatively correlated with kidney injury-related parameters and the Wnt/β-catenin signaling pathway transcripts, while positively correlated with tight junction proteins in the colon. The relative abundance of these beneficial bacteria was also negatively correlated with inflammatory factors not only in the colon but also in the kidney. By contrast, the relative abundance of dominant bacteria (such as *Rikenella, Prevotellaceae_UCG-001* and *Lachnoclostridium*) in the HS group had a strong positive correlation with injury severity in the colon and kidney.

Tax4Fun prediction analysis was then used to annotate the 16S rDNA data with metabolic pathways from the KEGG database (Fig. 3H). The relative abundance of the metabolism-associated signal transduction pathway showed a significant downregulation of the Wnt signaling pathway in the PLR-H group compared with that of the HS group, which was consistent with the GSEA results obtained from RNA-seq about the protective role of high-dose PLR in kidney injury. In addition, the vascular endothelial growth factor (VEGF) signaling pathway, which was proven by previous studies to promote fibrosis in CKD (38), was lower in the PLR-H group than that in the HS group, suggesting that fibrotic signaling pathways were suppressed by high-dose PLR supplementation.

Collectively, PLR administration could rectify gut microbiota dysbiosis and enhance the relative abundances of beneficial bacteria in mice, which were significantly negatively correlated with CKD severity.

### Gut microbiota reestablished by PLR reduce renal tissue fibrosis and intestinal epithelial barrier impairment

**Figure 4.**
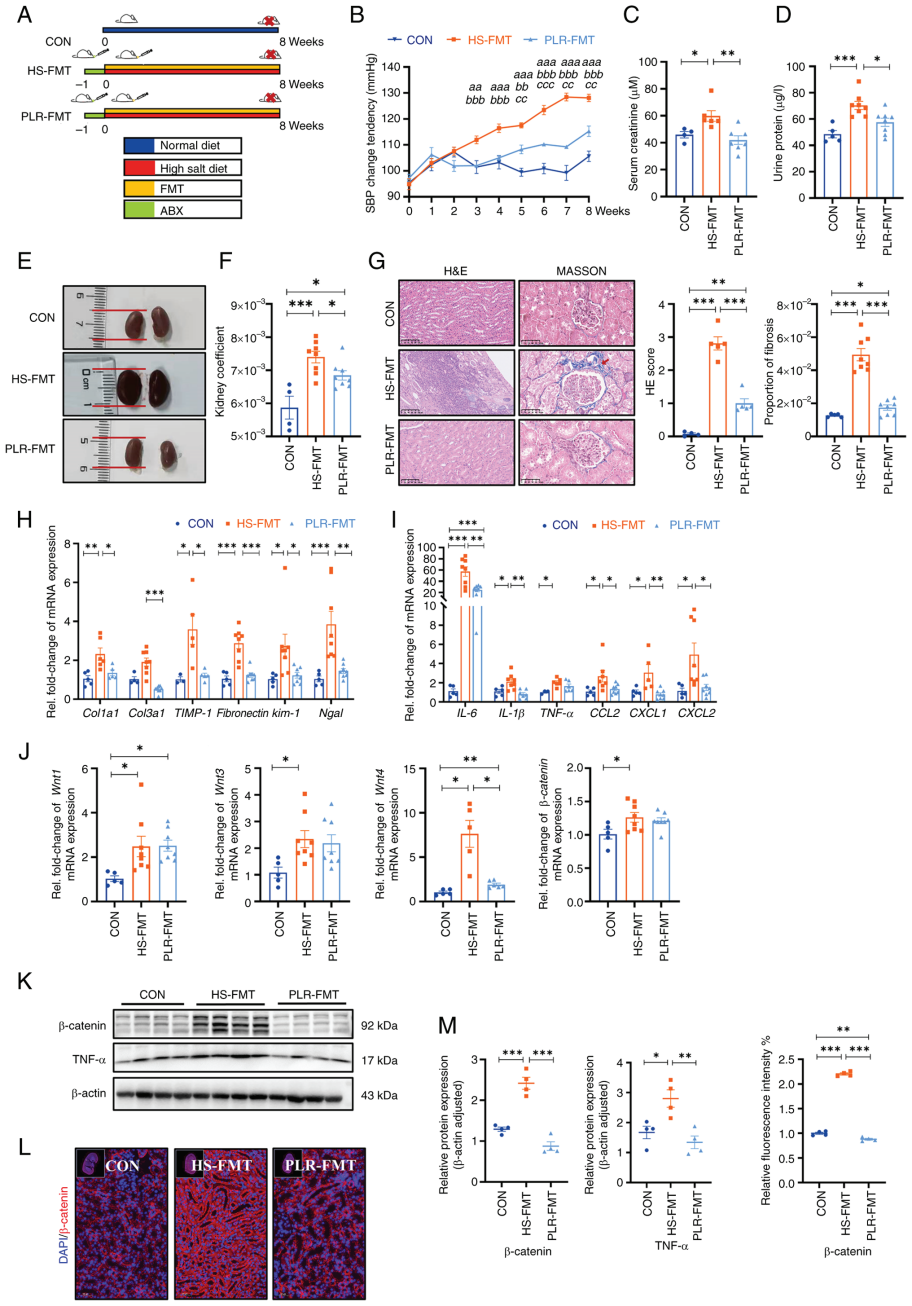
Gut microbiota from mice treated with PLR improve kidney tissue damage induced by HS diet and downregulate the Wnt/β-catenin pathway. (A) Flow chart of FMT experimental design. 8-week-old male C57BL/6 mice were administered drinking water containing 2% w/v NaCl for 8 weeks. (B) SBP change was evaluated non-invasively during experiment. ^aa^P<0.01 and ^aaa^P<0.001 for the CON group vs. the HS-FMT group on the corresponding week; ^bb^P<0.01 and ^bbb^P<0.001 for the PLR-FMT group vs. the HS-FMT group on the corresponding week; ^cc^P<0.01 and ^ccc^P<0.001 for the PLR-FMT group vs. the CON group on the corresponding week. Two-way repeated-measures ANOVA with Bonferroni's post hoc test was used for statistical analysis. (C) Serum creatinine after 8 weeks. (D) Urine protein after 8 weeks. (E) Representative gross anatomy pictures of the kidney. (F) Kidney coefficient. (G) H&E and MASSON staining of the kidney and the pathology scores and histological analysis., Fibrosis in the glomeruli and renal interstitium is indicated with a red arrow. H&E staining scale bar, 100 µm; MASSON staining scale bar, 50 µm (n=4-8). (H) RT-qPCR analysis of kidney injuries-related genes. (I) RT-qPCR analysis of inflammatory factors' genes in kidney. (J) RT-qPCR analysis of *Wnt1, Wnt3, Wnt4* and *β-catenin* genes in kidney (n=5-8). (K) β-catenin and TNF-α protein levels in the kidney. (L) Representative *in situ* detection of β-catenin in renal cortex was measured by immunofluorescent staining. Kidney tissue sections were stained with DAPI (blue) and probed with β-catenin (red). Scale bar, 50 µm (n=4). (M) Quantification of western blot and immunofluorescence. Results are expressed as the mean ± SEM (n=5–8 for each group). ***P<0.001, **P<0.01 and *P<0.05 were determined by one-way ANOVA with Bonferroni's post hoc test in C, D and F-I. PLR, *Puerariae lobatae* Radix; HS, high salt; FMT, fecal microbiota transplantation; ABX, antibiotic; SBP, systolic blood pressure; HE, hematoxylin-eosin; RT-qPCR, reverse transcription-quantitative PCR.

Next, to confirm whether the protective effect of PLR against CKD was dependent on the gut microbiome, the gut microbiota derived from mice treated with PLR-H were transplanted to CKD mice (Fig. 4A). Baseline weight and blood pressure had no differences among the groups (Fig. S4A-C). Compared with the HS-FMT group, SBP, serum creatinine and urine protein were significantly reduced in the PLR-FMT group (Fig. 4B-D). As presented in Fig. 4E and F, the PLR-FMT group demonstrated slight macroscopic injury and a lower kidney coefficient than that of the HS-FMT group; however, no statistical differences were exhibited in the liver and spleen coefficient among the three groups (Fig. S4D). Additionally, the kidney histopathological injury and fibrosis proportion were also reduced in the PLR-FMT group compared with those of the HS-FMT group (Fig. 4G). Furthermore, the mRNA levels of the inflammatory factors and chemokines, along with the mRNA levels of renal tubular-related injury and interstitial fibrosis, were also decreased in the PLR-FMT group compared with those of the HS-FMT group (Figs. 4H, I and S4E).

In addition, the activation of the Wnt/β-catenin pathway in the kidney was also explored. As shown in Fig. 4J, the mRNA levels of *Wnt1, Wnt3, Wnt4* and *β-catenin* of the PLR-FMT group were lower than those in the HS-FMT group. Moreover, compared with the HS-FMT group, the protein levels of β-catenin and TNF-α were significantly reduced in the PLR-FMT group (Fig. 4K-M). Taken together, these results suggested that the gut microbiota remodeled by PLR could protect kidney function, the effect of which is probably associated with the downregulation of the Wnt/β-catenin pathway.

**Figure 5.**
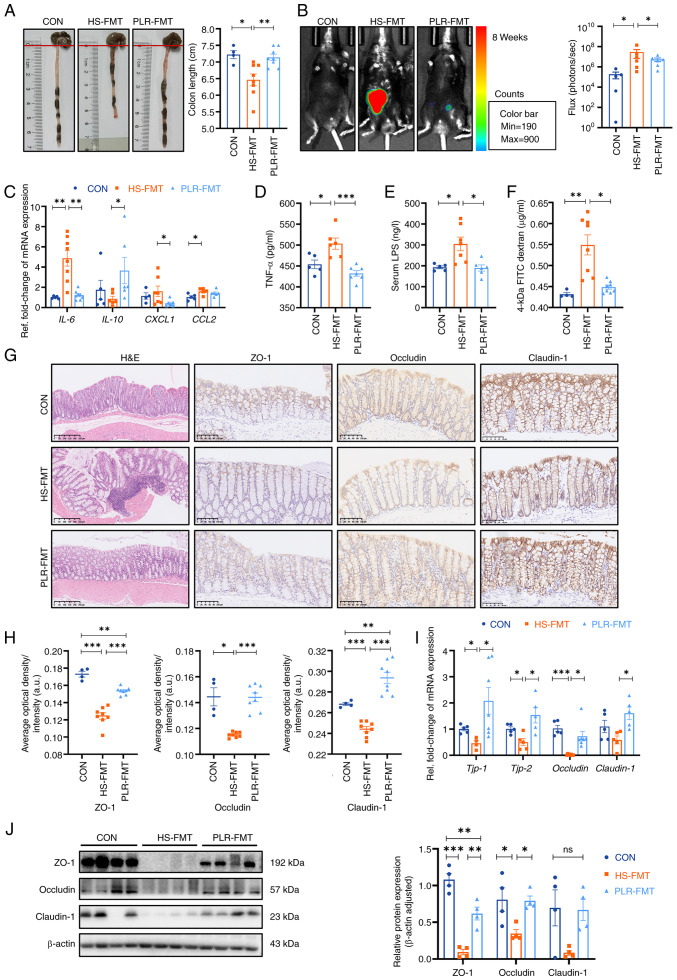
FMT from mice treated with PLR reduce intestinal inflammation and protect intestinal barrier function. (A) Representative gross anatomy pictures of the colon and the colon length measurement. (B) Representative L-012 fluorescent staining and animal fluorescence imaging (n=5-6). (C) RT-qPCR analysis of inflammatory factors' genes in colon. (D) Relative serum TNF-αlevels. (E) Relative serum LPS levels. (F) FD-4 levels in the plasma. (G) H&E staining and ZO-1, Occludin and Claudin-1 immunohistochemical staining in colon tissues. Scale bar, 100 or 250 µm (n=3-6). (H) Quantification of immunohistochemistry. (I) RT-qPCR analysis of tight junction proteins genes in colon (n=5-6). (J) ZO-1, Occludin and Claudin-1 protein levels in the colon (n=4). Results are expressed as the mean ± SEM (n=4–8 for each group). ***P<0.001, **P<0.01 and *P<0.05 were determined by one-way ANOVA with Bonferroni's post hoc test. FMT, fecal microbiota transplantation; PLR, *Puerariae lobatae* Radix; HS, high salt; CON, control; LPS, lipopolysaccharide; FD-4, FITC-dextran 4-KD; H&E, hematoxylin-eosin; ns, not significant; RT-qPCR, reverse transcription-quantitative PCR; ZO-1, zonula occludens 1.

Afterwards, it was investigated whether the gut microbiota derived from the PLR group was effective in alleviating the intestine inflammatory response and protecting intestinal barrier function. Colonic length was significantly reduced in the HS-FMT group compared with the CON group; however, this reduction was significantly mitigated by the intervention of PLR-derived gut microbiota and was restored to a length that was not significantly different from that of the CON group (Fig. 5A). Decreased mRNA levels of *IL-6, CXCL1* and *CCL2*, as well as increased *IL-10* mRNA expression in the colon were observed in the PLR-FMT group (Fig. 5C) compared with those of the HS-FMT group, which indicated a lower colonic inflammatory environment generated by PLR-derived gut microbiota. Furthermore, the bioluminescence imaging system exhibited images of less ROS in the colonic tissues of the PLR-FMT group than that of the HS-FMT group (Fig. 5B). Furthermore, H&E staining of the PLR-FMT colon revealed less abscission and epithelial cell necrosis, and less inflammatory cell infiltration compared with that of the HS-FMT group. As presented in Fig. 5G-J, the mRNA and protein levels of ZO-1, Occludin and Claudin-1 in the colon were significantly higher; while the serum TNF-α level, LPS level and FD-4 permeability were significantly lower in the PLR-FMT group than those in the HS-FMT group (Fig. 5D-F). These results suggested that the HS-derived gut microbiota triggered gut permeability by deteriorating intestinal barrier function, while the microbiota remodeled by PLR protected against colonic inflammation and intestinal barrier disruption, which reduced translocation of bacterial products and inflammatory cytokines into the serum. In addition, the aforementioned results indicated that the gut microbiota reestablished by PLR could reduce renal tissue fibrosis and colonic epithelial barrier damage.

### Gut microbiota rebuilt by PLR promote intestinal homeostasis in CKD

**Figure 6.**
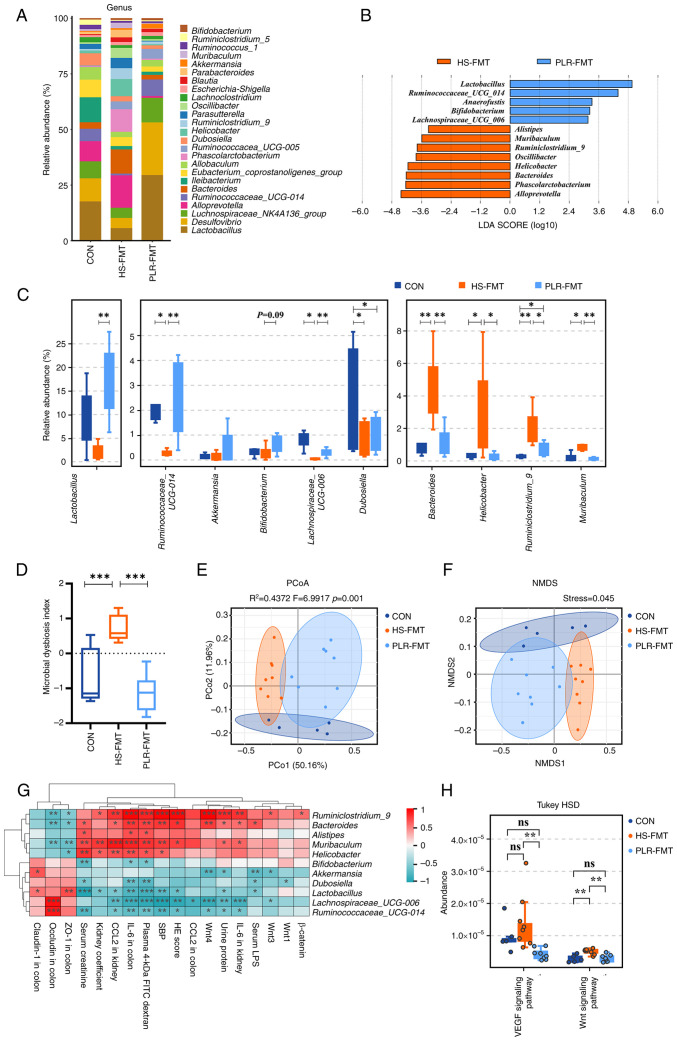
FMT from mice treated with PLR relieve intestinal microbial dysbiosis and rebuild healthy microbiota environment. (A) Relative bacterial abundance at the genus level in the feces of mice. (B) Histogram of the LDA score showing the biomarker at the genus level between the HS-FMT group and the PLR-FMT group. (C) Relative abundance of indicator species at the genus level showing the enriched bacteria in the gut microbiome among groups. (D) Microbial Dysbiosis index of each group. (E) PCoA based on the weighted UniFrac analysis of operational taxonomic units. (F) NMDS based on the weighted UniFrac analysis of operational taxonomic units. (G) Correlation heatmap of major indicator species and biomarkers based on LDA score and major injury indicators, scale shows correlation coefficient. (H) Kyoto Encyclopedia of Genes and Genomes pathway analysis of function distribution and difference analysis based on Tax4Fun prediction results. Results are expressed as the mean ± SEM (n=5–8 for each group). ***P<0.001, **P<0.01 and *P<0.05 were determined by one-way ANOVA with Bonferroni's post hoc test or Kruskal-Wallis test with Dunn's post hoc test in C and D, adonis analysis and anosim analysis in E, Spearman analysis in G, ANOVA test with Tukey's HSD test in H. FMT, fecal microbiota transplantation; HS, high salt; PLR, *Puerariae lobatae* Radix; LDA, linear discriminant analysis; PCoA, principal coordinates analysis; NMDS, non-metric multidimensional scaling.

To investigate the gut microbiota characteristic that exerted renoprotective effects in the FMT intervention, the microbial compositions from the HS-FMT and PLR-FMT groups were further analyzed. The main composition in either the phylum or genus level was similar among the groups (Figs. 6A and S5A). Notably, LEfSe and indicator species analysis indicated that *Lactobacillus* and *Ruminococcaceae*_UCG_014 at the genus level were significantly enriched by PLR-FMT (LDA score >3.6, Fig. 6B and C), while higher relative abundances of *Akkermansia* and *Bifidobacterium* were also found in the PLR-FMT group. As shown in Fig. S6A, the main composition in the genus level was similar between the PLR-H and the PLR-FMT groups. Indicator species analysis revealed 111 shared genera species in the PLR-H and PLR-FMT groups (Fig. S6B). There were no significant differences in the relative abundance of major genera. The relative abundance of *Akkermansia* and *Alistipes* in the PLR-FMT group was significantly lower than that of the PLR-H group, but the relative abundance of *Lactobacillus* and *Lachnoclostridium* was significantly higher than that of the PLR-H group (Fig. S6C). In addition, the increased MDI value in the HS-FMT mice was reversed by PLR-FMT intervention (Fig. 6D). Although alpha diversity was not impacted by either HS-FMT or PLR-FMT administration (Fig. S5B), PCoA analysis and NMDS showed that the gut microbiota structure was completely clustered (Fig. 6E and F). Furthermore, Spearman correlation analysis revealed that *Lactobacillus, Akkermansia* and *Ruminococcaceae*_UCG_014, which were enriched in the PLR-FMT group, were significantly positively correlated with the gut barrier-related tight junction protein, while negatively correlated with damage-related parameters and the Wnt signaling pathway-related genes in kidney (Fig. 6G). Finally, different gut microbial metabolic functions were explored via Tax4Fun prediction analysis from the KEGG data (Fig. 6H). The VEGF and Wnt signaling pathways were significantly downregulated in PLR-FMT mice compared with the HS-FMT group.

Overall, the aforementioned results indicated that FMT intervention could alter the structure of the gut microbiota, and the supplementary microbiota remodeled by high-dose PLR exerted a similar renoprotective effect as the PLR-H group. Further, *Lactobacillus, Akkermansia* and *Bifidobacterium* might be the dominating beneficial bacteria that induced CKD alleviation in both the PLR-H and PLR-FMT groups.

## Discussion

PLR is a traditional Chinese medicine according to the Chinese pharmacopoeia, and its main components are puerarin, daidzin and daidzein. These three main components are flavonoid natural drug compounds, which are characterized by low solubility, poor absorption and rapid metabolism, which to a moderate extent limit their application in diseases. However, PLR is not a simple superimposition of the aforementioned three individual ingredients. It has been previously shown that compared with the administration of pure daidzin alone, administration of the same dose of crude daidzin contained in a methanol extract of Radix *Puerariae* increased bioavailability of daidzin by 10-fold (39). It can be observed that PLR solves the dilemma of low oral bioavailability but high bioactivity of monomer drugs. In the present study, for the first time, to the best of our knowledge, it was found that oral PLR administration could alleviate CKD. On the other hand, from the point of view of intestinal flora, it is more likely to be affected by PLR compared with monomer components. The results of preclinical and clinical studies on herb-microbiota interactions suggest that traditional herbs can exert health-promoting and disease-preventing effects by influencing the structure of the intestinal flora (40). In the present study, the protective effect of PLR was achieved, at least partly, by remodeling the gut microbiota.

Salt is one of the most common dietary elements and plays an important role in maintaining the water-salt balance of the body. In the present study, a prolonged excessive HS diet led to gut microbiota disruption, intestinal inflammation and permeability increasement. It was also identified that an HS diet induced both inflammatory and fibrotic damage to the kidneys, probably because of harmful bacterial metabolites, such as LPS and inflammatory factors, including TNF-α, originating from the gut that then enters the circulation and induces damage to distant organs. However, the therapeutic intervention of PLR could protect against both intestinal and renal injury, which may be a result of its ability to reverse disturbances in the gut microbiota. It was also found that PLR intervention increased *Akkermansia, Lactobacillus* and *Bifidobacterium* and decreased *Rikenella, Prevotellaceae_UCG-001* and *Lachnoclostridium*. Previously, numerous clinical studies have found that the gut microbiota can mediate associations between the gut and other organs, such as via the ‘gut-brain axis’ and ‘gut-kidney axis’ (41,42). This suggests that the gut microbiota may be a potential target for intervention in abenteric disease progression. Indeed, it was confirmed that PLR exerted efficacy, at least partially, through the gut microbiota in the FMT experiment. *Akkermansia muciniphila* (*A. muciniphila*) is considered one of the key players in colonic mucus-associated microbiota and is necessary for the gut to produce mucus to maintain a healthy mucus layer and intestinal wall thickness (43,44). A recent study demonstrated that increased colonization of *A. muciniphila* in the colon of mice mitigated gut barrier leakage and blood endotoxemia in experimental colitis (45). Furthermore, previous clinical research revealed that the relative abundance of *Akkermansia* in patients with CKD was significantly lower than that in the healthy group (46). Oral gavage of mice with *A. muciniphila* protected against HFD/CCl_4_-induced liver and kidney fibrosis by modulating the inflammatory response (47). Moreover, *A. muciniphila* administration suppressed epithelial-mesenchymal transition and reduced renal interstitial fibrosis in 5/6 nephrectomy rats (48). This suggested that besides repairing the intestinal barrier, *Akkermansia* was also closely associated with alleviating the damage of CKD and renal fibrosis. Similarly, in the present study, the results also revealed that the relative abundances of *Akkermansia* were negatively correlated with the nephritic histopathological fibrotic degree and biochemical indexes of kidney injury. Therefore, it was hypothesized that the alleviating effect of PLR on CKD and renal fibrosis may be associated with increased colonization of *Akkermansia* in the intestine.

The gut microbiota is a complex ecosystem in which various bacteria strains can interact with each other through resource competition and nutrient symbiosis (14). The protective effect of a single bacterium, the symbiotic relationship between beneficial bacteria in intestinal homeostasis maintenance and disease protection should not be overlooked (49). In the present study, it was found that PLR also increased the relative abundances of *Bifidobacterium* and *Lactobacillus*, which are capable of enhancing the intestinal mucus layer and goblet cell function, thus protecting intestinal barrier integrity (50,51). Additionally, *in vitro* experiments demonstrated that *A. muciniphila* can stimulate the growth and change the gene expression profile of *Lactobacillus* (52,53). Studies have also shown that *Bifidobacterium* and *Lactobacillus* have potential benefits in reducing uremic toxin levels and protecting against CKD (54–56). Regarding PLR, it is rich in macromolecules such as starch, cellulose and lignin (49,57). These macromolecules reach the colon and provide energy for the synergistic growth of carbohydrate-utilizing bacteria such as *Bifidobacterium* and *Lactobacillus* (9,14,58). In addition, the aforementioned probiotics, together with *Faecalibaculum*, which could be enriched by PLR intervention in the present study, have been reported as SCFA-producing bacteria (59–61), capable of increasing SCFA production and thus creating an anti-inflammatory and anti-oxidative environment in the intestine. Moreover, a non-inflammatory stable state reduced gut barrier damage and permeation of harmful metabolites into the plasma, thus decreasing the adverse impact on abenteric organs. Taken together, the gut microbiota reshaped by PLR and characterized by *Akkermansia, Bifidobacterium* and *Lactobacillus* is a key factor in its enteroprotective and nephroprotective effects.

A continuous intake of HS necessitates salt to be excreted in the urine to maintain the water-salt balance in the plasma, and this sustained kidney stimulation in urine production may cause pathological damage and kidney fibrosis. Currently, the Wnt signaling pathway is more accepted to influence HS-related CKD (62). Previous literature indicated that transient activation of Wnt/β-catenin facilitates kidney tissue generation after acute kidney injury, whereas sustained activation stimulates kidney fibrosis in CKD (63). With the presence of a HS load, mice presented with more fibrosis and upregulated Wnt/β-catenin signaling in the heart and kidney (64,65). In the present study, PLR intervention significantly alleviated the degree of renal fibrosis in HS-induced CKD. More importantly, GSEA showed the downregulation of the canonical Wnt signaling pathway in the PLR group. This might explain a potential pathway by which PLR protects the kidney from fibrosis. In the present study, the Tax4Fun prediction analysis from the KEGG database was inspected and it was found that the Wnt signaling pathway was correlated with the gut microbial metabolism. Besides, the current results showed that the activation of the Wnt/β-catenin pathway and the level of pathological damage in the kidney were significantly negatively correlated with the relative abundance of beneficial bacteria, such as *Akkermansia, Lactobacillus* and *Bifidobacterium*, increased by PLR intervention, which led the authors to hypothesize that the gut microbiota may be effective in regulating the Wnt signaling pathway expression and subsequently decelerating renal fibrosis progression. Numerous studies have found that the Wnt signaling pathway can be regulated by the gut microbiota (66,67). Treatment of mice with *A. muciniphila* is reported to significantly suppress epithelial-mesenchymal transition and reduce renal interstitial fibrosis (48). Furthermore, treatment of *Bifidobacterium bifidum* and *Lactobacillus gasseri* with quercetin could inhibit the canonical Wnt/β-catenin signaling pathway to protect against colorectal cancer in mice (68). Both PLR and PLR-FMT interventions could significantly downregulate the Wnt signaling pathway. From this, it was hypothesized by the authors that alterations in the Wnt signaling pathway and alleviation of renal fibrosis by PLR intervention was associated with the gut microbiota and its metabolism.

Inflammation and other signaling pathways can also contribute to CKD. In the present study, a significantly elevated TNF-α protein level was found in the serum and kidneys of CKD mice. Increased TNF-α is one of the upstream targets for triggering β-catenin activation in the kidney (69,70), and the reduction of β-catenin has been shown to result in a lower expression of fibrosis markers including fibronectin, Col1a1 and Col3a1 (71–74). However, the elevation of TNF-α and β-catenin was significantly suppressed by PLR intervention in the present study, which added to the possible targets of the nephroprotective effects of PLR. In addition, the VEGF signaling pathway could also be downregulated by PLR intervention in the current study. Previous studies have shown that numerous therapies targeted the HIF-1α/VEGF signaling pathway to relieve liver fibrosis (75,76). The VEGF signaling pathway was also reported to have a synergistic effect with the Wnt/β-catenin signaling pathway in angiogenesis and fibrosis (77,78). In the present study, these two pathways may have corporate effects on renal fibrosis after PLR intervention.

In the present study, the protective effect of PLR against elevated blood pressure induced by HSD was found. Sodium excretion and diuresis using diuretics is one of the clinical strategies to reduce blood pressure. In the present study, there was a trend toward higher urinary sodium excretion in the PLR group compared with the HS group, but the difference was not significant. To the best of our knowledge, no previous studies have identified a diuretic effect of PLR or its main components. Therefore, further exploration is needed regarding whether PLR can promote urinary sodium excretion.

PLR, a traditional Chinese medicine and food homologous herb, has multi-target and multi-pathway pharmacodynamic routes of action. In the present study, it was confirmed that PLR could alleviate CKD by remodeling the gut microbiota, repairing the intestinal epithelial barrier, and downregulating the Wnt signaling pathway in the kidney. However, there are certain limitations to the present study; although a previous study suggested that removal of gut flora could attenuate HS diet-induced kidney injury (11), in the present study, this finding was not validated and the FMT experiment was directly performed. Furthermore, the present study focused on gut microbiota entirely and its beneficial effect on CKD, and single strains of bacteria were not accessed. In addition, although focus was addressed on Wnt1, Wnt3 and Wnt4 in the canonical Wnt/β-catenin signaling pathway, this does not mean that other Wnt genes have no influence (79). It has been previously revealed that upregulation of the Wnt4 and Wnt5 genes could activate the non-canonical Wnt pathway in hepatic stellate cells of fibrotic livers (80). Moreover, it was apparent that the beneficial pharmaceutical effects of PLR were mediated through several mechanisms. Previous studies have shown that mTOR and AMPK pathways are also involved in the regulation and progression of CKD (81,82). In the future, more in depth understanding about the remodeling ability of PLR towards the gut microbiota should be further explored and mTOR and AMPK pathways could be potential targets for subsequent research. Overall, the present study provided evidence for a new function of PLR regarding kidney protection and a novel direction for the treatment of kidney disease.

## Supplementary Material

Supporting Data

Supporting Data

## Data Availability

The data generated in the present study may be found in the Genome Sequence Archive (Genomics, Proteomics & Bioinformatics 2021), National Genomics Data Center (Nucleic Acids Res 2022), China National Center for Bioinformation/Beijing Institute of Genomics, Chinese Academy of Sciences under accession numbers CRA015711, CRA015797 and CRA015712 or at the following URL: https://ngdc.cncb.ac.cn/gsa.
