# Supplementary material for: Puerariae lobatae Radix ameliorates chronic kidney disease by reshaping gut microbiota and downregulating Wnt/β‑catenin signaling
Source: Mol Med Rep. 2024 May 14;30(1):117. doi: 10.3892/mmr.2024.13241 (PMC11129539; doi:10.3892/mmr.2024.13241)

Figure S1. Chromatographic peaks of PLR samples analyzed for composition by ultra performance liquid chromatography. Testosterone acted as the internal standard. (A) Testosterone; (B) puerarin; (C) daidzein; (D) daidzin. PLR, *Puerariae lobatae* Radix.

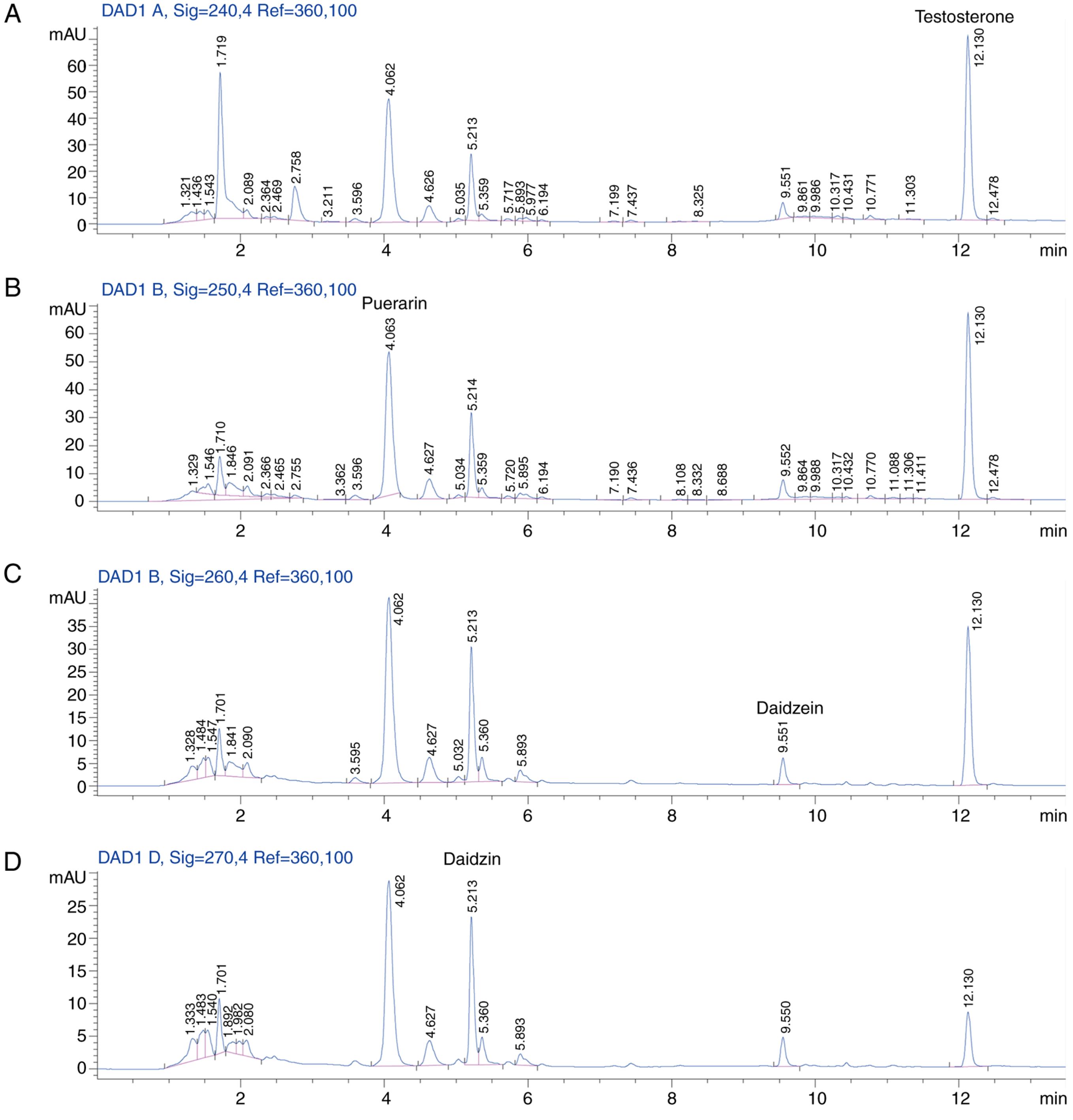

Figure S2. Baseline measurement and change of mice in the whole process of experiment. (A) Baseline weight (n=6). (B) Baseline SBP (n=6). (C) Baseline MBP (n=6). (D) MBP change of groups was evaluated non-invasively during experiment (n=6). (E) Weight change of groups (n=6). $^{aa}P<0.01$ and $^{aaa}P<0.001$ for the CON group vs. the HS group on the corresponding week. $^{c}P<0.05$ and $^{cc}P<0.01$ for the PLR-H group vs. the CON group on the corresponding week. (F) Liver coefficient (n=6). (G) Spleen coefficient (n=6). (H) Average water intake of each mouse during experiment (n=6). (I) Serum K^+ concentration (n=6). (J) Serum Ca^{2+} concentration (n=6). (K) Differential analysis of gene expression between HS and PLR group (n=3). Results are expressed as the mean ± SEM. Statistical analysis was performed using one-way ANOVA with Bonferroni's post hoc test in A-C, F, G and I-J, and two-way repeated-measures ANOVA with Bonferroni's post hoc test in D and E. The differential gene filtration threshold is false discovery rate <0.05, |log2FC|≥1 in K. PLR, *Puerariae lobatae* Radix; HS, high salt; SBP, systolic blood pressure; MBP, mean blood pressure; ns, not significant.

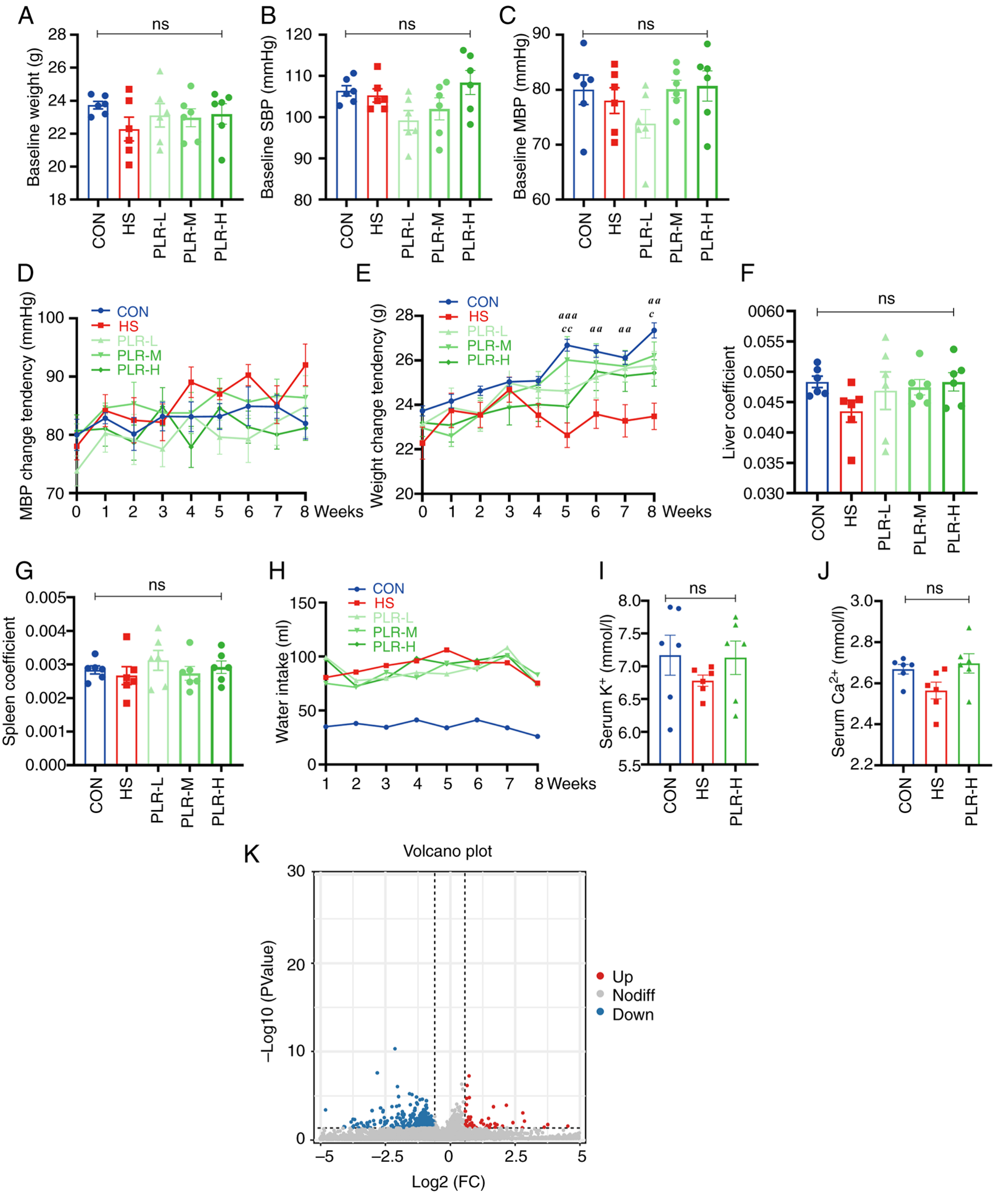

Figure S3. PLR affects the composition of gut microbiota in HS mice and reverses intestinal dysbacteriosis. (A) Relative amounts of Bacteroidetes, Firmicutes, Firmicutes/Bacteroidetes ratio and Verrucomicrobia in the cecal content. (B) Relative bacterial abundance at the phylum level in the feces of mice. (C) Alpha diversity indices. (D) Correlation between the mRNA levels of inflammatory factors and tight junction proteins in the colon and the abundance of *Akkermansia* and *Lactobacillus* in the feces of mice. Results are expressed as the mean ± SEM (n=6-10 for each group). ***P<0.001, **P<0.01 and *P<0.05 were determined by one-way ANOVA with Bonferroni's post hoc test in A and C, Spearman analysis in D. PLR, *Puerariae lobatae* Radix; HS, high salt; ns, not significant.

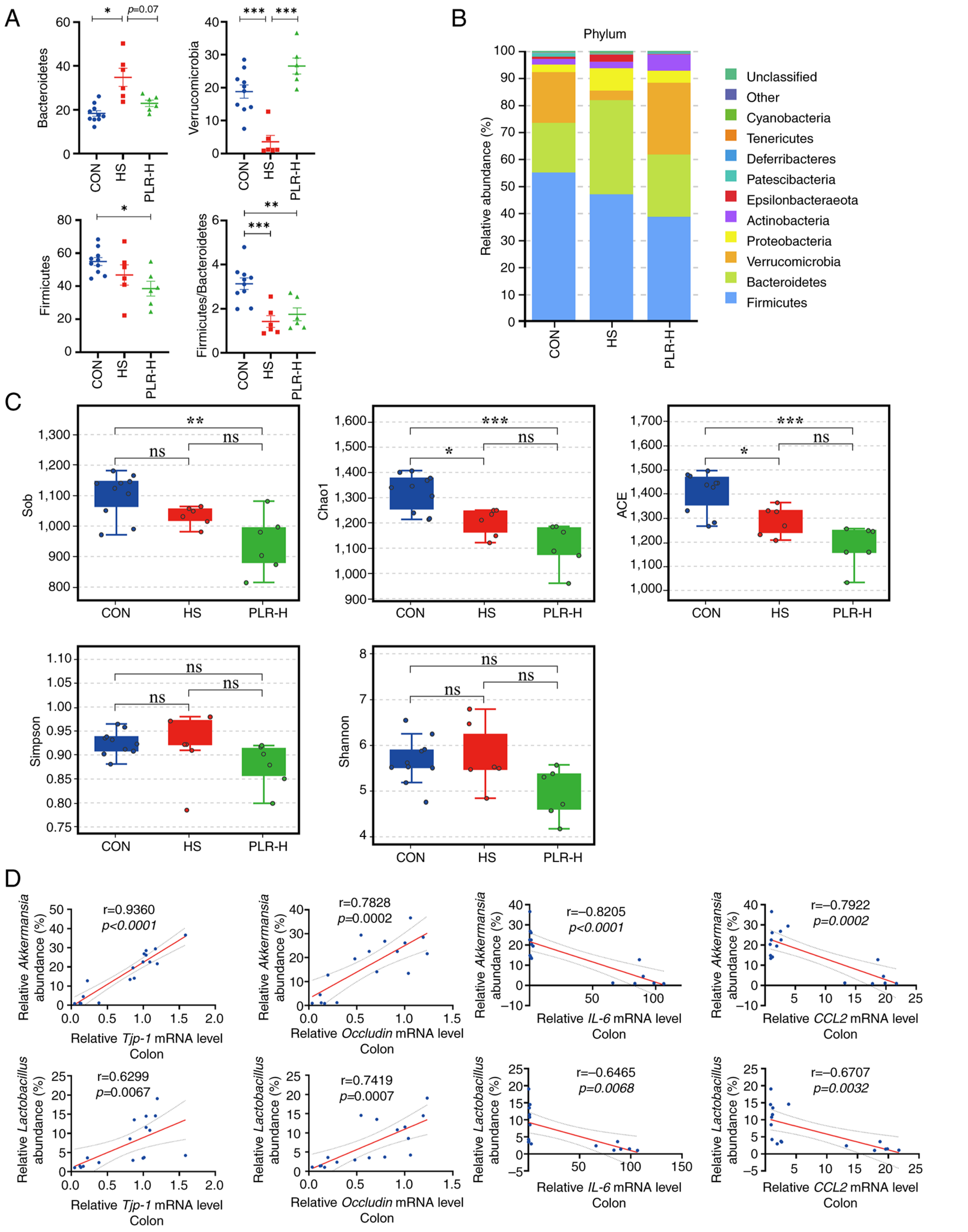

Figure S4. Baseline measurement and change of mice in the whole process of FMT experiment. (A) Baseline weight (B) Baseline SBP. (C) Baseline MBP. (D) Liver coefficient and spleen coefficient. (E) Reverse transcription-quantitative PCR analysis of *IL-10* and *F4/80* genes in kidney. Results are expressed as the mean ± SEM (n=4-8 for each group). *P<0.05 was determined by one-way ANOVA with Bonferroni's post hoc test. PLR, *Puerariae lobatae* Radix; HS, high salt; FMT, fecal microbiota transplantation; SBP, systolic blood pressure; MBP, mean blood pressure; ns, not significant.

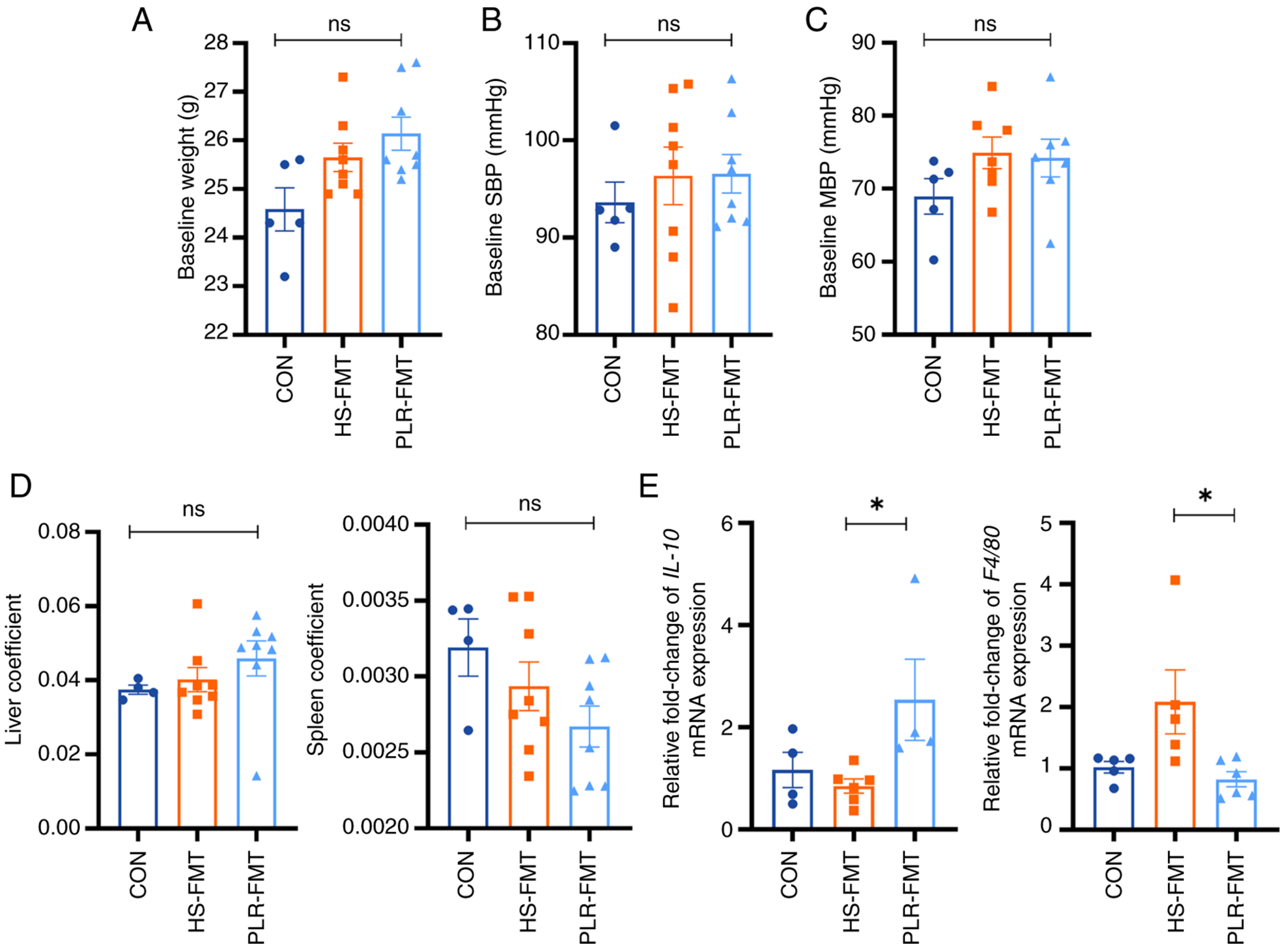

Figure S5. FMT from mice treated with PLR affect the composition of gut microbiota in HS mice and reverse intestinal dysbacteriosis. (A) Relative bacterial abundance at the phylum level in the feces of mice. (B) Alpha diversity indices. Results are expressed as the mean ± SEM (n=5-8 for each group). $^{*}P<0.05$ was determined by one-way ANOVA with Bonferroni's post hoc test or Kruskal-Wallis test with Dunn's post hoc test in B. FMT, fecal microbiota transplantation; PLR, *Puerariae lobatae* Radix; HS, high salt; ns, not significant.

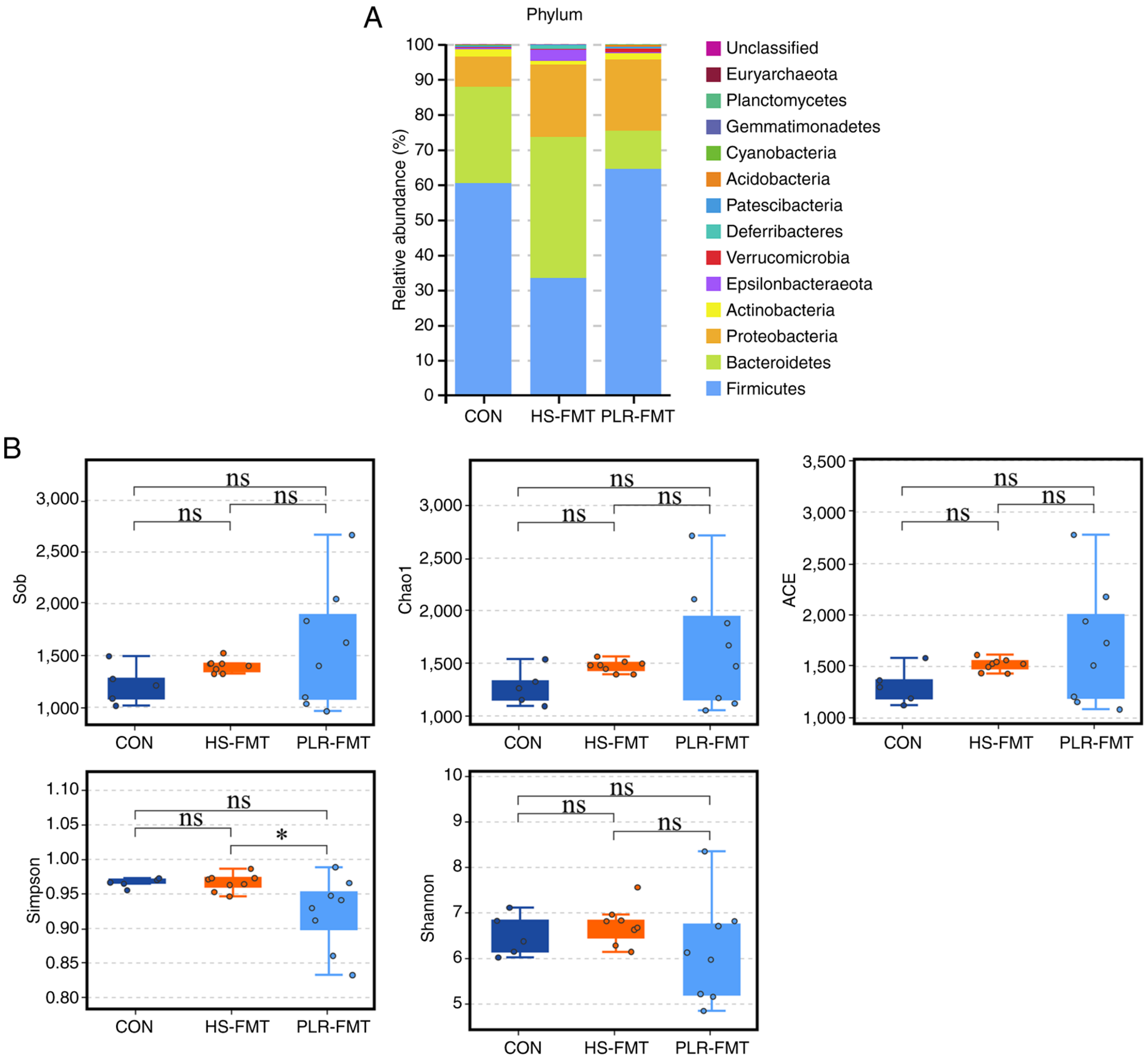

Figure S6. FMT from mice treated with PLR affect the composition of gut microbiota. (A) Relative bacterial abundance at the genus level in the feces of mice. (B) Analysis of indicator species at the genus level between groups. (C) Relative abundance of major bacteria at the genus level between groups (n=6-8 for each group). ***P<0.001 and **P<0.01 were determined by Mann-Whitney test in C. FMT, fecal microbiota transplantation; PLR, *Puerariae lobatae* Radix; ns, not significant.

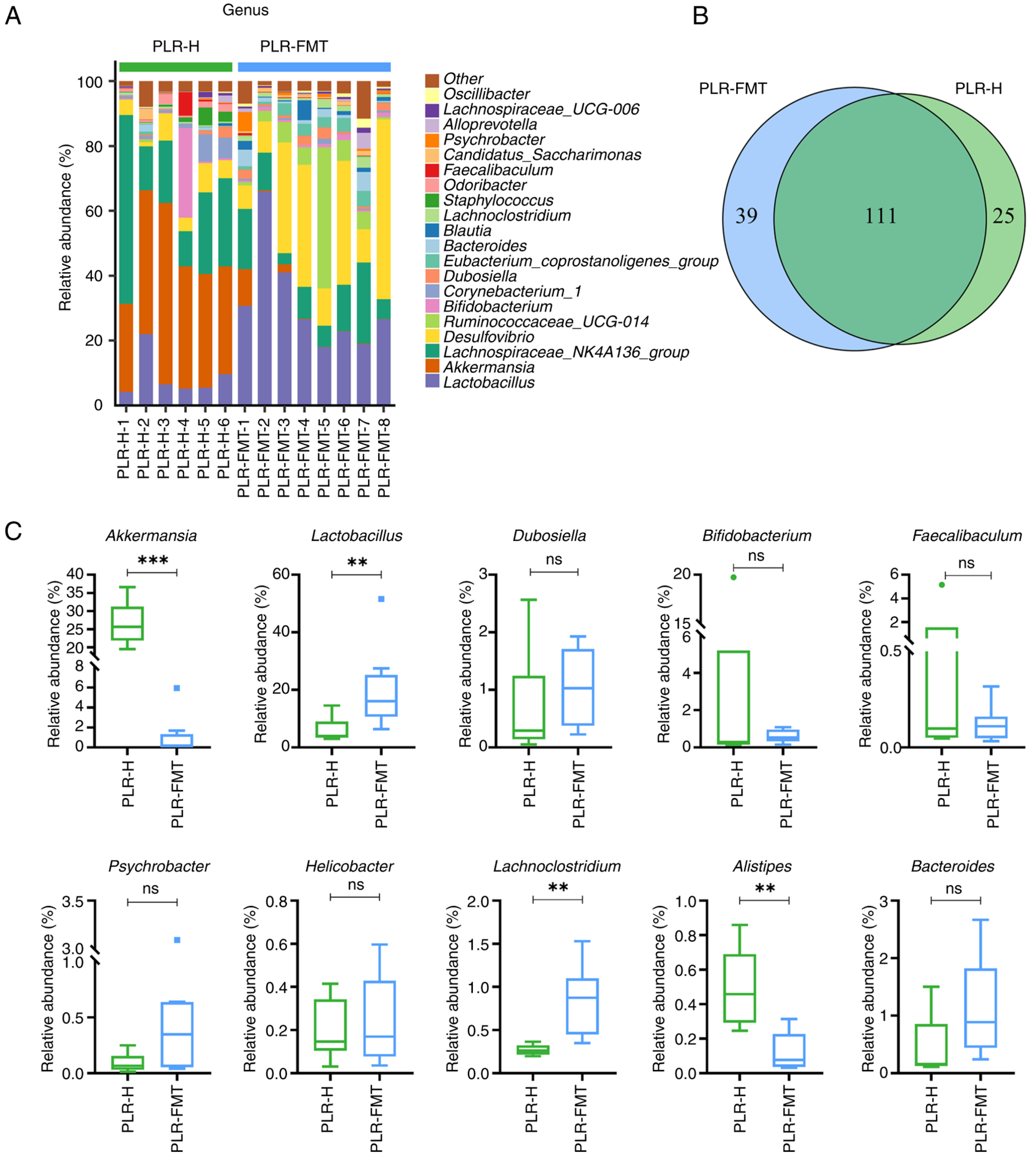

Supplement: Supporting Data [file Supplementary_Data1.pdf]
