# Supplementary material for: Puerariae lobatae Radix ameliorates chronic kidney disease by reshaping gut microbiota and downregulating Wnt/β‑catenin signaling
Source: Mol Med Rep. 2024 May 14;30(1):117. doi: 10.3892/mmr.2024.13241 (PMC11129539; doi:10.3892/mmr.2024.13241)
Supplement: Supporting Data [file Supplementary_Data2.pdf]

Table SI. Changes in the proportion of mobile phase.

Time (min)	Water (%)	Methanol (%)
0.00	67	33
0.00	67	33
3.00	67	33
3.50	50	50
7.50	50	50
8.00	20	80
13.00	20	80
13.50	67	33

The column was an Agilent ZORBAX SB-C18 (3.0 x 100 mm, 1.8-Micron). The mobile phase comprised methanol and water with a flow rate of 0.3 ml/min. Puerarin, daidzin and daidzein were detected at a UV wavelength of 250, 270 and 260 nm, respectively. Testosterone (240 nm) acted as the internal standard. The retention times of puerarin, daidzin and daidzein were 3.82, 5.19 and 9.55 min, respectively.

Table SII. Primer sequences for quantitative PCR.

Gene name	Primer sequence (5'-3')
Col1a1	F: CGGAGAAGAAGGAAAACGAGGAG
	R: CACCATCAGCACCAGGGAAAC
Col3a1	F: GCACAGCAGTCCAACGTAGA
	R: TCTCCAAATGGGATCTCTGG
TIMP-1	F: GCAACTCGGACCTGGTCATAA
	R: CGGCCCGTGATGAGAAACT
Fibronectin	F: GTGGCTGCCTTCAACTTCTC
	R: TGAATGCCAGTCCTTTAGGG
Kim-1	F: ACATATCGTGGAATCACAACGAC
	R: ACTGCTCTTCTGATAGGTGACA
Ngal	F: GCAGGTGGTACGTTGTGGG
	R: CTCTTGTAGCTCATAGATGGTGC
IL-6	F: GAGCCCACCAAGAACGATAG
	R: TCCACGATTTCCCAGAGAAC
TNF-α	F: AGGGTCTGGGCCATAGAACT
	R: CCACCACGCTCTTCTGTCTAC
CCL2	F: TTAAAAACCTGGATCGGAACCAA
	R: GCATTAGCTTCAGATTTACGGGT
CCL3	F: CAGCCAGGTGTCATTTTCCT
	R: CTCAAGCCCCTGCTCTACAC
Wnt1	F: TCAGAACCGCAGCACAGAAC
	R: TTCACGATGCCCCACCATC
Wnt3	F: GGGGCGTATTCAAGTAGCTG
	R: GTAGGGACCTCCCATTGGAT
Wnt4	F: CGAGCAATTGGCTGTACCTGG
	R: CAGGCCTTTGAGTTTCTCG
β-catenin	F: GTGCAATTCCTGAGCTGACA
	R: CTTAAAGATGGCCAGCAAGC
IL-10	F: TGCTATGCTGCCTGCTCTTA
	R: TCATTTCCGATAAGGCTTGG
CXCL1	F: GCTGGGATTCACCTCAAGAA
	R: AGGTGCCATCAGAGCAGTCT
Claudin-1	F: GCCATCTACGAGGGACTGTG
	R: CCCCAGCAGGATGCCAATTA
Claudin-7	F: TGTACAAGGGGCTCTGGATG
	R: GGACACCACCATTAAGGCTC
Occludin	F: ACTCCTCCAATGGCAAAGTG
	R: CCCCACCTGTCGTGTAGTCT
Tjp-1	F: AGAGACAAGATGTCCGCCAG
	R: TGCAATTCCAAATCCAAACC
Tjp-2	F: GTGATTTTCTTCAACCCGGA
	R: TTTTTGAGCTTGTTGGCTTG
IL-1β	F: CAGGCAGGCAGTATCACTCA
	R: AGCTCATATGGGTCCGACAG
CXCL2	F: TCCAGAGCTTGAGTGTGACG
	R: CTTTGGTTCTTCCGTTGAGG
F4/80	F: TCTGGGGAGCTTACGATGGA
	R: GAATCCCGCAATGATGGCAC
GAPDH	F: AGGTCGGTGTGAACGGATTTG
	R: TGTAGACCATGTAGTTGAGGTCA

F, forward; R, reverse.
